# Antileukemic activity of the VPS34-IN1 inhibitor in acute myeloid leukemia

**DOI:** 10.1038/s41389-020-00278-8

**Published:** 2020-10-22

**Authors:** Godelieve Meunier, Rudy Birsen, Clarisse Cazelles, Maya Belhadj, Lilia Cantero-Aguilar, Olivier Kosmider, Michaela Fontenay, Nabih Azar, Patrick Mayeux, Nicolas Chapuis, Jerôme Tamburini, Didier Bouscary

**Affiliations:** 1 Institut Cochin, Université de Paris, CNRS UMR8104, INSERM U1016, Paris, France; 2 Equipe Labellisée Ligue Nationale Contre le Cancer (LNCC), Paris, France; 3 Service d’hématologie Clinique, Hôpital Cochin, APHP, Paris, France; 4 Service d’hématologie Biologique, Hôpital Cochin, APHP, Paris, France; 5 Service d’hémobiothérapie, Hôpital La Pitié Salpétrière, APHP, Paris, France; 6 Proteomic core facility of Paris Descartes University (3P5), Paris, France

**Keywords:** Target identification, Acute myeloid leukaemia

## Abstract

Acute myeloid leukemia (AML) is an aggressive disease with a poor prognosis. Vacuolar protein sorting 34 (VPS34) is a member of the phosphatidylinositol-3-kinase lipid kinase family that controls the canonical autophagy pathway and vesicular trafficking. Using a recently developed specific inhibitor (VPS34-IN1), we found that VPS34 inhibition induces apoptosis in AML cells but not in normal CD34+ hematopoietic cells. Complete and acute inhibition of VPS34 was required for the antileukemic activity of VPS34-IN1. This inhibitor also has pleiotropic effects against various cellular functions related to class III PI3K in AML cells that may explain their survival impairment. VPS34-IN1 inhibits basal and l-asparaginase-induced autophagy in AML cells. A synergistic cell death activity of this drug was also demonstrated. VPS34-IN1 was additionally found to impair vesicular trafficking and mTORC1 signaling. From an unbiased approach based on phosphoproteomic analysis, we identified that VPS34-IN1 specifically inhibits STAT5 phosphorylation downstream of FLT3-ITD signaling in AML. The identification of the mechanisms controlling FLT3-ITD signaling by VPS34 represents an important insight into the oncogenesis of AML and could lead to new therapeutic strategies.

## Introduction

Acute myeloid leukemia (AML) is an aggressive disease caused by the transformation of hematopoietic progenitor cells due to acquired genetic alterations^1^. Although new therapies for AML have emerged in recent years, the prognosis remains poor and new therapeutic strategies are needed^2^.

Vacuolar protein sorting 34 (VPS34) is a member of the phosphatidylinositol-3-kinase lipid kinase family. VPS34 binds to a regulatory subunit (VPS15) to form the only class III PI3K present in mammalian cells. This class III PI3K uses phosphatidylinositol (PIP) as a substrate to produce PI3P. PI3P then recruits proteins containing PI3P-recognizing domains such as FYVE, PX, and PROPPINS, which are involved in intracellular vesicular trafficking. Class III PI3K acts in the assembly of various complexes, allowing spatial and temporal control of PI3P production^3^. Thus, VPS34 is crucial for key cellular functions such as autophagy, endocytic sorting, phagocytosis, and cell signaling^4,5^.

Autophagy is a catabolic process that drives the uptake of cytoplasmic constituents to lysosomes, where they are degraded and recycled. From an oncogenic perspective, autophagy has differential impacts in distinct phases of tumorigenesis^6^. In healthy cells, autophagy constitutes a barrier against malignant transformation. In neoplastic cells however, autophagy sustains survival and proliferation upon exposure to intracellular and environmental stress, hence supporting tumor growth, invasion, and metastatic dissemination^6^. The deregulation of autophagy has been reported in AML^7–10^. Historical and new treatments have been shown to induce autophagy, which may be protective or participate in cell death depending on the compound^11–14^. The use of autophagy inhibitors, either alone or in combination with other therapies, has emerged as a therapeutic approach to this disease^15–18^.

In recent years, chemical optimization has enabled the identification of specific VPS34 inhibitors^19–22^. One of these new inhibitors, VPS34-IN1, is a bis-aminopyrimidine that targets the hydrophobic region of the kinase ATP binding domain. Considering the role of autophagy in AML and the importance of VPS34 in this intracellular process, we investigated the antileukemic activity of VPS34 inhibition in our current study.

## Materials and methods

### Primary human samples

Bone marrow (BM) or peripheral blood (PB) samples with a >70% blast cells content were obtained from 23 patients with newly diagnosed AML (patient characteristics are provided in Supplemental Table 1). The CD34+ fraction enriched in hematopoietic progenitor cells (HPCs) was purified from allogenic bone marrow donors using MIDI MACS immunoaffinity columns (Milteny Biotech, Germany). Patients and healthy donors provided written informed consent in accordance with the Declaration of Helsinki and approval was obtained from the Cochin Hospital Institutional Ethic Committee.

### Cell lines and reagents

HL60, MOLM-14, MV4-11, OCI-AML2, OCI-AML3, U937, K562, THP1, and KASUMI AML cell lines were used (descriptions are provided in Supplemental Table 2). All AML cell lines were certified using their microsatellite identity and tested for mycoplasma contamination. Cells were cultured in RPMI (Gibco61870, Life Technologies® Saint Aubin, France) and supplemented with 10% fetal bovine serum (FBS) and 4 mM glutamine. VPS34 IN-1 was sourced from MRCC-PU Reagents (Dundee, Scotland). DMSO, chloroquine and doxycycline were obtained from Sigma-Aldrich (St. Louis, MO). Autophinib, PIK-III, Ferrostatine-1, Q-VAD-OPH, and necrostatine-1 were purchased from Selleckchem (Munich, Germany). Lysotracker deep red was obtained from Thermo Fischer Scientific (Asnières, France). l-Asparaginase was provided by Cochin hospital pharmacy Department.

### CRISPR/Cas9 genome editing

VPS34-specific RNA guides were designed using the Optimized CRISPR Design application from the laboratory of Dr. Feng Zhang (http://crispr.mit.edu/). We retained two guides: VPS34#1: GGATATCAACGTCCAGCTTA and VPS34#2: CTACATCTATAGTTGTGACC. The following nontargeted guide was used as a control (CTR): GTAGGCGCGCCGCTCTCTAC. These guides were then cloned into the plentiCRISPR plasmid (Addgene plasmid 49535)^23^ allowing the subsequent lentiviral infection of MOLM-14 cells.

### Constructs

The GFP-LC3 lentivirus was obtained from Merck (17-10193, LentiBrite™ GFP-LC3 Lentiviral Biosensor; Darmstat, Germany). The VPS34 shRNA plasmid was a gift from Dr Stephane Manenti (Cancer Research Center of Toulouse, INSERM). The construction of mammalian expression plasmids for Fms-like Tyrosine kinase 3 (FLT3) mutants was performed as previously described^24^.

### Single-cell clone selection

MOLM-14 cells were cultured in the presence of puromycin for one week after lentiviral infection for CRISPR targeting of the VPS34 gene. Single-cell cloning was then done using a FACSARIA II cell sorter (Becton Dickinson, Franklin Lakes, NJ). After two weeks, the cultured clones were analyzed by western blotting for VPS34 expression and by Sanger sequencing of the amplicons surrounding the target sequence on VPS34 exon 1. The PCR and sequencing primers were as follows: left primer, TCCTGTACCTAAGTTCCCGC; right primer, AGCAATCCCACTCCCTGTC.

### Flow cytometry-based assay for autophagy

Flow cytometry-based assays for autophagy were performed as described previously^25^. Briefly, GFP-LC3 expressing cells were washed once with PBS and then with either PBS containing 0.05% saponin or with PBS alone. Ten thousand events were captured for analysis. Flow cytometry data were collected using a C6 Accuri flow cytometer (Becton Dickinson, Le Pont de Claix, France) with CFlow Plus software. Data analysis was then carried out with CFlow Plus software (Becton Dickinson, Le Pont de Claix, France). Data acquisition and data analysis were conducted at the Cochin Cytometry and Immunobiology Facility.

### Western blotting

Preparation of whole-cell extracts and western blotting were performed as previously described^11^. The antibodies used are detailed in Supplemental Table 3.

### Electron microscopy

Cells were fixed with paraformaldehyde 2% and glutaraldehyde 3.75%. After several thorough washes in PBS buffer, the cells were post-fixed in 1% osmium tetroxide and embedded in epoxy resin. Semithin sections of 300–400 nm were first realized to control the samples and 80–90 nm sections were then cut on a Reichert Ultracut E ultramicrotome. These ultrathin sections were next transferred onto 200-mesh copper grids prior to staining with uranyl acetate and lead citrate. The sections were then viewed under a JEOL 1011 transmission electron microscope with a GATAN Orius 1000 CCD camera.

### Immunofluorescence

MOLM-14 EGFP-LC3 cells (2 × 10^5^) were fixed in methanol after cytocentrifugation. The cells were then visualized on a Zeiss inverted microscope and LC3 dots were automatically quantified using ImageJ with a dedicated built-in Macro (Thomas Guibert, IMAGI’C, Cochin institute).

### Viability assay

AML cells were plated at 10 × 10^4^/ml in 100 μl volumes (1 × 10^3^ cells per well) of 10% FBS-supplemented RPMI prior to the addition of VPS34-IN1. Cells were cultured in the presence of VPS34-IN1 5 µM for 48 h at 37 °C. Viability was quantified using the fluorescence-based Uptiblue assay (Interchim, Montluçon, France). Uptiblue was added in 10 μl aliquots to each well. Fluorescence was measured with a Typhoon FLA9500 scanner (GE Healthcare; Chicago, IL). Fluorescence values were normalized to DMSO-treated controls for each AML cell line. IC_50_ values were calculated using four parameters nonlinear regression with Graph Pad Prism v8 (GraphPad, La Jolla, CA).

### Phosphoproteomic analysis

Phosphoproteomic analysis, performed on MOLM-14 cells cultured with vehicle or VPS34-IN1 is detailed in the Supplemental Materials and Methods. The mass spectrometry proteomics data have been deposited to the ProteomeXchange Consortium via the PRIDE partner repository with the dataset identifier PXD016839.

### Proliferation assays

Cells were seeded at 10 × 10^4^/ml on day 0 and counted manually using trypan blue staining after 3 days.

### Apoptosis assay

Apoptosis was quantified by flow cytometry via staining with annexin V-PE (Becton Dickinson Biosciences, Le Pont De Claix, France) or TMRE (ab113852 Abcam, Paris, France).

### Measurement of synergistic effects

Cell viability was calculated for every dose combination of VPS34-IN1 and l-Asparaginase using the Synergy Finder web tool (https://synergyfinder.fimm.fi/) in comparison to each agent alone. Calculations were done against the ZIP model^26^.

### Statistics

Differences between the mean values obtained for the experimental groups were analyzed using the two-tailed Student’s *t*-test. Statistical analyses were performed using Prism software (GraphPad, La Jolla, CA). *p ≤ 0.05, **p ≤ 0.01, ***p ≤ 0.001, ****p ≤ 0.0001.

## Results

### Anti-leukemic activity of the VPS34 IN1 inhibitor in AML

**Fig. 1:**
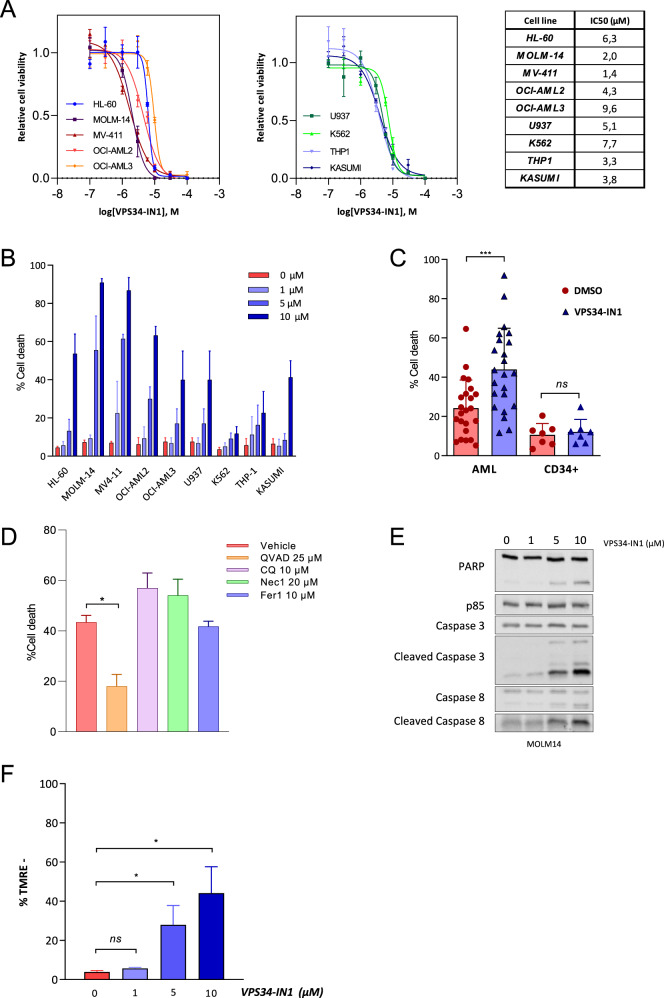
The VPS34 IN1 inhibitor has antileukemic activity against AML. **A** AML cell lines were cultured for 48 h in the presence of VPS34-IN1 over a large concentration range and viability was quantified using the fluorescence-based Uptiblue assay. This enabled a determination of the IC_50_ values for VPS34-IN1; *n* = 3, error bars represent the standard deviation. **B** AML cell lines were cultured for 48 h with vehicle or 1, 5, or 10 µM of VPS34-IN1. Cell death was quantified by flow cytometry analysis of the percentage of annexin-V positive cells; *n* = 3, bars represent the standard error of the mean. **C** Primary AML cells from 23 newly diagnosed AML patients (Supplemental Table 1) were treated for 48 h with vehicle or 5 µM VPS34-IN1. Normal CD34+ hematopoietic progenitors from 6 allogenic bone marrow donors were cultured under similar conditions after positive sorting. Cell death was quantified by the percentage of annexin-V positive cells. Bars represent standard error of the mean. **D** Cell death (%) was quantified by flow cytometry analysis of the percentage of annexin-V positive cells for MOLM14 cells at 24 h post-VPS34-IN1 treatment (5 µM) with vehicle or ferrostatine-1 (10 µM), necrostatine-1 (20 µM), chloroquine (20 µM) or QVD-oph (25 µM); *n* = 3, errors bars represent the standard deviation. All other compounds were added to the medium 2 h before VPS34-IN1. **E** MOLM-14 cells were cultured for 48 h with vehicle or VPS34-IN1 at 1, 5, or 10 µM and western blot analysis was performed using antibodies directed against PARP, caspase-3, cleaved caspase-3, caspase-8, cleaved caspase-8, or p85. **F** MOLM-14 cells were cultured for 48 h with vehicle or VPS34-IN1 at 1, 5, or 10 µM and TMRE staining was performed to assess mitochondrial depolarization; *n* = 3, bars represent the standard error of the mean.

We tested the antileukemic activity of the VPS34-IN1 compound in nine AML cell lines. VPS34-IN1 impaired viability and induced dose-dependent cell death in all these tested cell lines (Fig. 1A, B). We then tested the effects of VPS34-IN1 on the survival of primary leukemic cells from 23 patients with AML. This inhibitor induced a significant death of leukemic cells in this series of AML patients (Fig. 1C and Supplemental Table 1). In contrast, VPS34-IN1 did not induce cell death in normal CD34+ hematopoietic progenitor cells from 6 allogenic BM donors (Fig. 1C). We next investigated the mechanisms of VPS34-IN1-induced cell death. MOLM-14 cells treated with VPS34-IN1 were exposed to inhibitors of various known cell death mechanisms (Fig. 1D). The pan-caspase inhibitor Q-VAD-OPH prevented the cell death response induced by VPS34-IN1 at 24 h. However, no other inhibitors of other forms of cell death, including autophagy (chloroquine), necroptosis (necrostatine-1) or ferroptosis (ferrostatine-1) could suppress VPS34-IN1-induced cell death. VPS34-IN1 was further found to induce the dose-dependent cleavage of PARP, caspase-3, and caspase-8 proteins (Fig. 1E) and dose-dependent mitochondrial depolarization, as revealed by TMRE staining (Fig. 1F). Taken together, these results demonstrated that VPS34 IN1 induces mitochondrial apoptotic cell death in AML cells.

### The complete and acute inhibition of VPS34 is required for VPS34-IN1 anti-leukemic activity

**Fig. 2:**
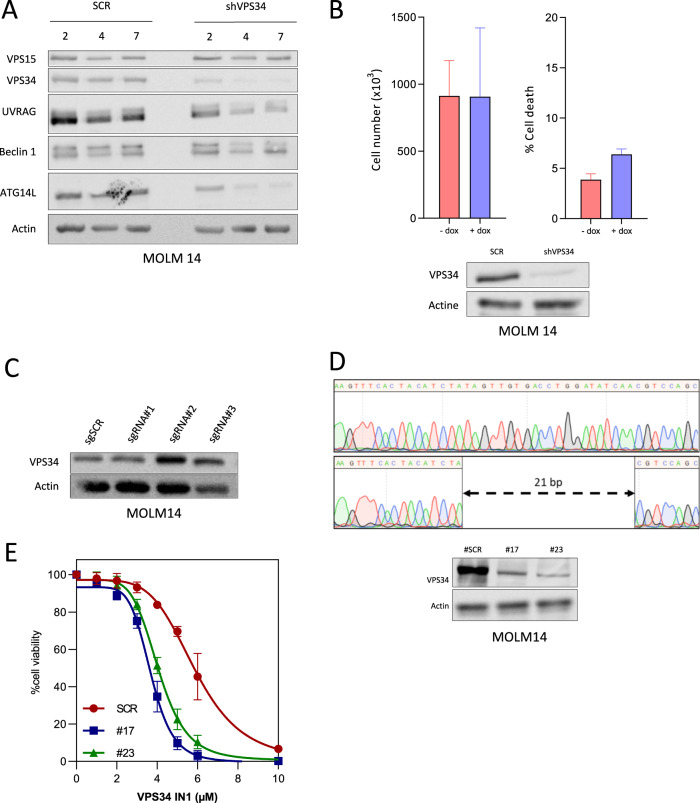
The complete and acute inhibition of VPS34 is required for VPS34-IN1 anti-leukemic activity. **A** MOLM-14 cells engineered to express a doxycycline-inducible VPS34 shRNA were cultured with vehicle or doxycycline for 2, 4, and 7 days. The expression of β-actin, VPS15, VPS34, UVRAG, beclin-1, and ATG14L was assayed by western blotting. **B** MOLM-14 cells expressing a VPS34 shRNA construct were cultured with vehicle or doxycycline and the cell number and cell death were quantified at 48 h. **C** VSP34 expression in MOLM-14 scrambled (SCR) cells or MOLM-14 cells with a disrupted expression of VPS34 induced using CRISPR/CAS9 technology (bulk) was assessed by western blot. **D** Upper panel: representative Sanger sequencing of VPS34 exon 1 in MOLM-14 SCR cells or MOLM-14 cells with disrupted VPS34 expression (subclone 17). Lower panel: western blot analysis of VSP34 expression in MOLM-14 SCR cells or MOLM-14 cells with disrupted VPS34 expression (subclones 17 and 23). **E** MOLM14 SCR and MOLM-14 cells with disrupted VPS34 expression (subclones 17 and 23) were cultured for 48 h in the presence of VPS34-IN1 over a large concentration range. Viability was quantified using the fluorescence-based Uptiblue assay; *n* = 3, bars represent standard error of the mean.

We generated MOLM-14 cells expressing a doxycycline-inducible VPS34 shRNA. The loss of VPS34 protein expression in these cells when cultured with doxycycline was partial at day 2 but was elevated to over 80% between days 4 and 7 (Fig. 2A). Disruption of the VPS34 complexes was observed in these cells with decreased expression of the associated proteins UVRAG and ATG14-L (Fig. 2A). However, a reduction in VPS34 expression did not suppress cell proliferation or induce apoptosis (Fig. 2B). Our shRNA strategy could not fully repress VPS34 expression, suggesting that complete and acute inhibition of VPS34 may be necessary for the antileukemic effects of the VPS34-IN1 compound. Indeed, it has been shown that a reduced VPS34 level poorly impairs its cellular functions, suggesting that compensatory mechanisms may play a role in maintaining cell viability in this instance^27–29^. Accordingly, MOLM-14 cells with a reduction in VPS34 could still induce autophagy, one of the key cellular mechanisms controlled by VPS34 (Supplemental Fig. 1A). Hence, we tried to generate MOLM-14 cells harboring a homozygous deletion of the VPS34 locus using CRISPR/Cas9 technology. No decrease in VPS34 was found in the bulk of infected cells for the three designed sgRNAs (Fig. 2C). Among the 288 subclones analyzed (96/sgRNA), only eight showed VPS34 protein downregulation (Supplemental Fig. 1B). All these subclones were from cells infected with sgRNA targeting exon 1. Homozygous or heterozygous deletions were observed in all these clones, but these deletions were consistently detected in the reading frame, as exemplified by clone 17, which was insufficient to completely abrogate protein expression (Fig. 2D). These results strongly suggested that a complete loss of VPS34 is not viable in AML cells, thus explaining our inability to obtain sub clones with a complete inhibition of VPS34 using CRISPR/CAS9 technology. However, the antileukemic activity of VPS34-IN1 was enhanced in clones 17 and 23 compared to the scrambled control which demonstrated a good specificity of VPS34-IN1 (Fig. 2E). To provide additional evidence for the specificity of the VPS34-IN1 compound, we asked if other VPS34 inhibitors could reproduce the anti-leukemic activity of VPS34-IN1. We used two others chemically distinct VPS34 inhibitors: autophinib^30^ and PIK-III^20^. Both inhibitors induced dose-dependent cell death in a panel of AML cell lines in micromolar range and the sensitivity profile of each cell line was superimposable to VPS34-IN1 (supplemental Fig. 2A, B). We also showed an inverse correlation between VPS34 protein expression and the sensitivity to VPS34 inhibition (by VPS34-IN1 or genetic depletion) in AML cell lines (Supplemental Fig. 3A–C). Altogether, these results showed that the anti-leukemic activity of VPS34-IN1 is related to specific inhibition of VPS34.

### VPS34-IN1 inhibits intracellular vesicle trafficking and basal autophagy in AML cells

**Fig. 3:**
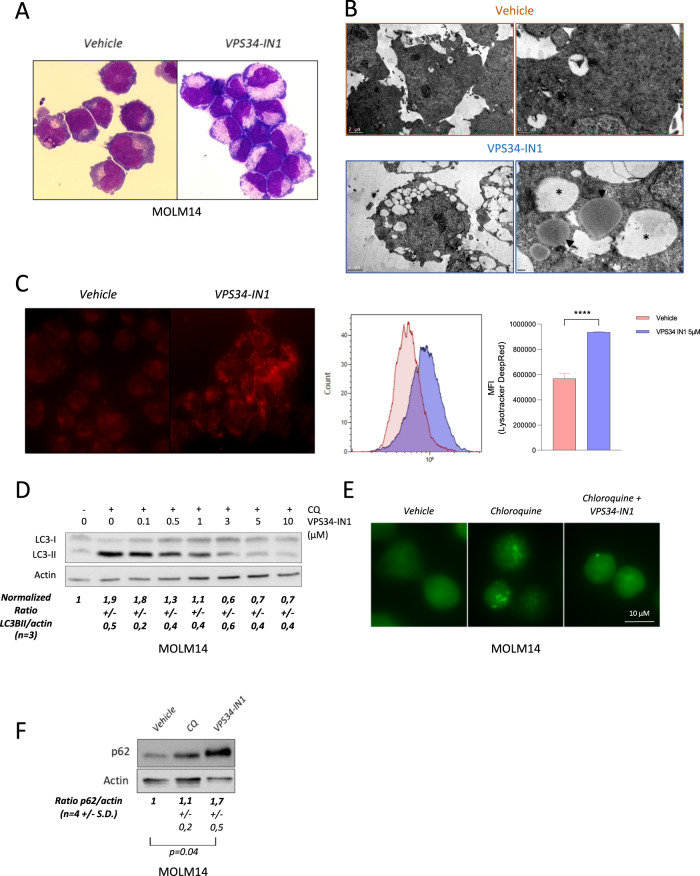
VPS34-IN1 inhibits intracellular vesicle trafficking and basal autophagy in AML cells. **A** MOLM-14 cells were cultured for 48 h with vehicle (left panel) or VPS34-IN1 (5 µM, right panel) and observed by optical microscopy (×100) after May Gruenwald Giemsa staining. **B** MOLM-14 cells were cultured for 72 h with vehicle (upper panel) or VPS34-IN1 (5 µM, lower panel) and observed by electron microscopy. Large-sized empty vacuoles were observed in VPS34-IN1 treated cells representing single-membraned swollen endosomes/lysosomes (*). Lipid droplets (arrowheads) were also observed in VPS34-IN1 treated cells. **C** MOLM-14 cells were cultured for 24 h with vehicle (left) or VPS34-IN1 (5 µM, right) and observed by immunofluorescence microscopy (×100) following Lysotracker deep red staining. MFI was also measured by FCM. **D** MOLM 14 cells were cultured for 6 h with vehicle or chloroquine (10 µM) and various doses of VPS34-IN1. Western blot analysis was then performed using antibodies directed against LC3-II and actin. **E** Representative immunofluorescence results of MOLM-14 GFP LC3 cells treated with vehicle, chloroquine alone (10 µM), or chloroquine with VPS34-IN1 (5 µM). **F** MOLM-14 cells were cultured for 24 h with vehicle, chloroquine (10 µM) or VPS34-IN (5 µM) and p62 accumulation was assessed by western blotting.

As class III PI3K is involved in multiple cellular functions, we sought to understand the molecular processes that could explain the antileukemic effects of VPS34-IN1. May Grunewald Giemsa examinations of cells exposed to VPS34 inhibitors (VPS34-IN1, PIK-III, and autophinib) revealed multiple intracellular vesicles (Fig. 3A and Supplemental Fig. 4A). This had been reported previously in a VPS34-KO model and is related to the blockage in the endocytic trafficking within the cell^27^. Using electronic microscopy and immunofluorescence analysis following Lysotracker deep red staining, we confirmed the accumulation of late-endosomes and lysosomes induced by VPS34 inhibitors in MOLM-14 cells (Fig. 3B, C and Supplemental Fig. 4B). As VPS34 controls the canonical autophagy pathway, we studied the effects of VPS34 inhibition on basal autophagy. As expected, VPS34-IN1 inhibited the basal autophagic flux (as indicated by the inhibition of LC3-II accumulation by chloroquine) in AML cells at a micromolar dose range, with complete inhibition achieved at around 5 µM (Fig. 3D). Autophinib and PIK-III were also able to inhibit this basal autophagic flux (Supplemental Fig. 4C). Accumulation of the GFP-LC3 was reduced in presence of VPS34-IN1 (Fig. 3E). Conversely, the protein level of p62 was increased (Fig. 3F). PIK-III similarly increased p62 levels in MOLM-14 and MV4-11 cell lines (Supplemental Fig. 4D). We thus concluded that VPS34 inhibition can strongly inhibit intracellular vesicle trafficking and basal autophagy in AML cells.

### VPS34-IN1 inhibits l-asparaginase-induced autophagy and both drugs show synergistic antileukemic activity

**Fig. 4:**
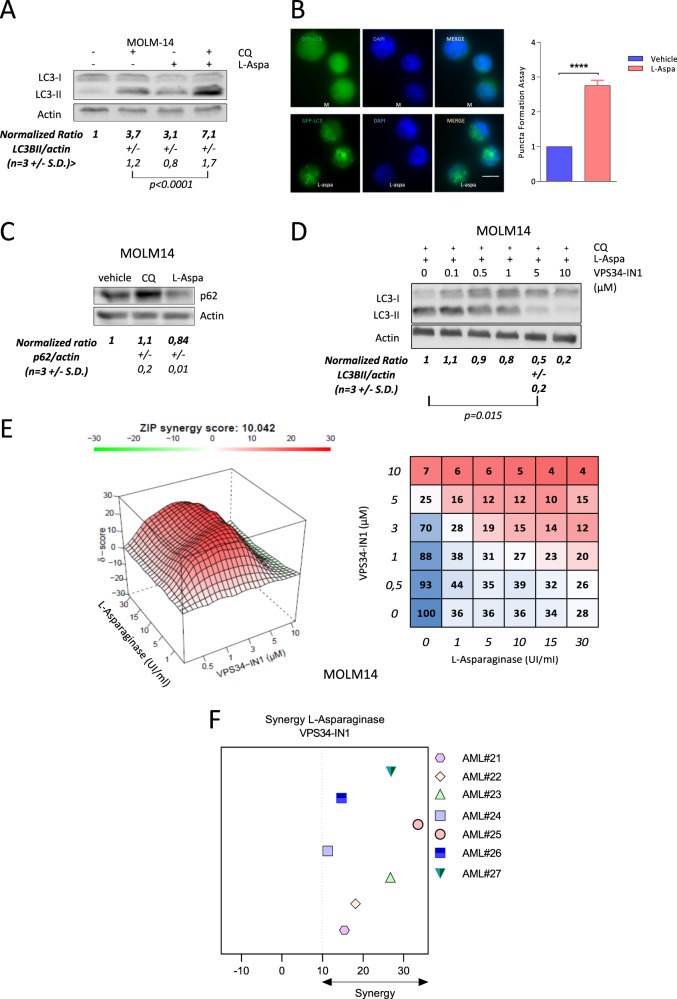
VPS34-IN1 inhibits l-asparaginase induced autophagy and bot drugs act synergistically. **A** MOLM-14 cells were cultured for 6 h with l-asparaginase and/or chloroquine (10 µM) as indicated. Autophagy induction was assessed by analyzing both LC3-I and LC3-II expression by western blot. Upon autophagic induction, LC3-I is transformed to LC3-II which is then degraded in autophagolysosome. Chloroquine is able to inhibit acidification of autophagolysosome, allowing the evaluation of LC3-II formation which is an indirect marker of autophagy induction **B** Number of LC3B puncta per cells (right) and immunofluorescence analysis of GFP LC3 (left) in vehicle-treated or l-Asparaginase-treated (10 UI/ml) MOLM-14 cells were determined; *n* = 3, bars represent standard error of the mean. **C** MOLM-14 cells were cultured for 24 h with vehicle, chloroquine (10 µM) or l-asparaginase (10 UI/ml) and p62 accumulation was assessed by western blot. **D** MOLM-14 cells were cultured with increasing concentrations of VPS34-IN1 and in the presence of both l-asparaginase (10 UI/ml) and chloroquine (10 µM). Western blotting analysis of the LC3-I and LC3-II bands was performed at 6 h. **E** Synergy map (left) and viability matrix (right) of l-asparaginase with VPS34-IN1 for the MOLM-14 cell line. The mean viability measurement from three independent experiments was used. **F** Summary of synergy score from 48 h co-treatments of seven primary AML samples with l-Asparaginase and VPS34-IN1.

We next determined whether VPS34-IN1 could efficiently modulate drug-induced autophagy. Previous data have suggested a strong induction of autophagy with the drug l-asparaginase in AML^11^ and that autophagy is required for cell survival under l-asparaginase-induced metabolic stress in acute lymphoblastic leukemia cells^31^. We first confirmed the induction of autophagy following exposure to this drug. Kinetic analysis of the expression of the LC3-II band in MOLM-14 cells treated with l-asparaginase revealed a time-dependent accumulation of the LC3-II form, suggestive of autophagy induction (Supplemental Fig. 5A). Analysis of the autophagic flux with chloroquine revealed that l-asparaginase promotes LC3-II band accumulation, attesting to a positive autophagic flux (Fig. 4A). l-asparaginase-induced autophagy was also detected by flow cytometry analysis of the fluorescence of MOLM-14 cells expressing a GFP-LC3 vector (Supplemental Fig. 5B). Immunofluorescence analysis of MOLM-14 GFP-LC3 cells at 6 h after l-asparaginase treatment detected significantly more GFP-fluorescent puncta (Fig. 4B). l-asparaginase was also able to induce an autophagic flux in 3 (AML 6; 8; 12) out of 4 primary AML samples tested (Supplemental Fig. 5C). As a result of autophagy induction, the p62 protein level decreased, due to its degradation in MOLM-14 cells treated with l-asparaginase (Fig. 4C). Taken together these results indicated that l-asparaginase induces strong autophagy in AML cells.

We studied the effects of VPS34-IN1 in association with l-asparaginase. VPS34-IN1 repressed the accumulation of the LC3-II form in MOLM-14 cells treated with l-asparaginase in a dose-dependent manner, with near complete inhibition achieved at 5 µM (Fig. 4D). In MOLM-14 cells and in most of the primary AML samples, this combination treatment induced a greater apoptotic response than either agent alone, suggesting a synergism in exerting antileukemic activity (Supplementary Fig. 5D). Synergistic effects were further demonstrated using the synergy finder website in MOLM-14 cells and in seven primary AML samples treated with increasing concentrations of both VPS34-IN1 and l-asparaginase (Fig. 4E, F and Supplemental Fig. 6). Overall, these results demonstrate that VPS34-IN1 is a potent inhibitor of l-asparaginase-induced autophagy and that an anti-leukemic synergy exists between l-asparaginase and VPS34-IN1.

### VPS34-IN1 modulates mammalian target of rapamycin complex 1 (mTORC1) and FLT3-ITD signaling in AML cells

**Fig. 5:**
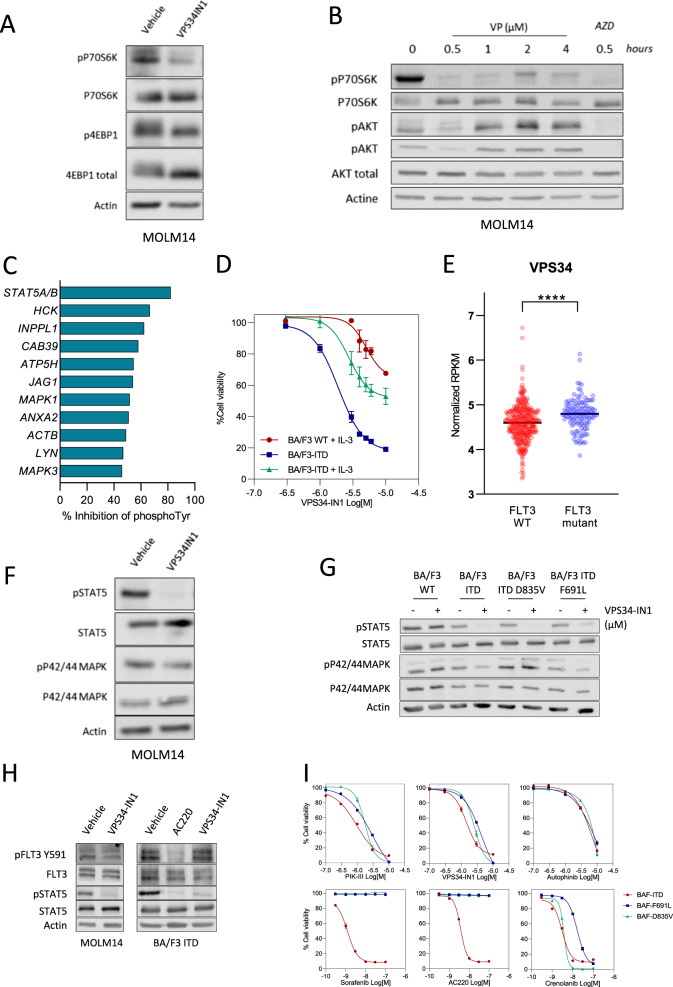
VPS34-IN1 modulates mTORC1 and FLT3-ITD signaling in AML cells. **A** MOLM-14 cells were cultured for 6 h with vehicle or VPS34-IN1 (5 µM). Western blot analysis was used to evaluate the activation of the mTORC1 pathway using Thr 37/46 p4EBP1 and Thr 389 pP70S6K antibodies. **B** MOLM-14 cells were cultured for 0–4 h with VPS34-IN1 (5 µM). Western blot analysis was used to study the activation of the mTORC1 pathway using a Thr389 pP70S6K antibody and PI3K/AKT pathway activation was assessed using antibodies directed against phosphorylated AKT Thr308 or AKT S473 residues. AZD8055, a TOR kinase inhibitor, was used as a control for the inhibition of the mTORC1 and AKT Ser473 signaling pathways. **C** Percentage inhibition of phosphotyrosine in the 11 most downregulated proteins identified by phosphoproteomic analysis. **D** BA/F3 WT and BA/F3 cells with FLT3-ITD expression, with or without IL-3, were cultured for 48 h in the presence of VPS34-IN1 over a large concentration range. Viability was quantified using the fluorescence based Uptiblue assay; *n* = 3, bars represent standard error of the mean. **E** Comparison of VPS34 expression (normalized RPKM) in AML cells from patients with or without FLT3 mutation. Data are from Vizome database, a part of the Beat AML project^36^. **F** MOLM-14 cells were cultured with vehicle or 5 µM VPS34-IN1 for 1 h. Western blotting was then used to analyze FLT3 expression, and STAT5 and P42/44MAPK phosphorylation. **G** Ba/F3 cells were cultured in the presence of IL-3 with vehicle or VPS34-IN1 (5 µM) for 3 h. Ba/F3 cells engineered to harbor either the FLT3-ITD, FLT3-ITD-TKD D835V or FLT3-ITD-TKD F691L mutations, which render them IL-3 independent for growth, were cultured under similar conditions. Western blot analysis was used to study the activation of the STAT5 pathway using a pSTAT5 antibody and MAPK1/3 pathway using a p42/44MAPK antibody. **H** MOLM-14 cells and BA/F3 cells expressing FLT3-ITD were cultured without or with 5 µM VPS34-IN1 for 1 h. The FLT3-ITD inhibitor AC220 was used as a positive control for the inhibition of FLT3 phosphorylation. Western blotting was used to analyze FLT3 expression, FLT3 phosphorylation and STAT5 pathway activation. **I** Ba/F3 cells were cultured in the presence of IL-3 with vehicle or various dose of VPS34-IN1, PIK-III, autophinib, sorafenib, AC220, and crenolanib for 48 h. Ba/F3 cells engineered to harbor either the FLT3-ITD, FLT3-ITD-TKD D835V or FLT3-ITD-TKD F691L mutations, which render them IL-3 independent for growth, were cultured under similar conditions. Viability was quantified using the fluorescence based Uptiblue assay; *n* = 3, bars represent standard error of the mean.

VPS34 can indirectly regulate the signaling mediated by several protein kinases through its function in the regulation of endosomal trafficking. Notably in this regard, the class III PI3K has been reported to be involved in the full activation of the mTORC1 complex^32–34^. The mTORC1 pathway is constantly overactivated in AML and constitutes a therapeutic target^35^. We thus investigated the impact of VPS34-IN1 on the activation of the mTORC1 complex in AML. VPS34-IN1 suppressed the phosphorylation of P70S6K Thr389 and 4EBP1 Thr37/46, indicating mTORC1 inhibition in MOLM-14 cells (Fig. 5A). mTORC1 inhibition resulted in the activation of the class I PI3K as shown by the increased phosphorylation of Akt on both Thr308 and Ser473 (Fig. 5B).

We next sought to identify proteins whose phosphorylation on tyrosine residues was modulated by VPS34-IN1, in order to detect new signaling pathways controlled by VPS34 in AML. We performed phosphoproteomic analysis to quantify the effects of VPS34-IN1 using the SILAC method (see “Methods” section, Supplemental data). Samples from two different experiments performed in MOLM-14 cells treated or not with VPS34-IN1 were analyzed. Overall, 7279 phosphorylation sites corresponding to 2616 different proteins were quantified. Two hundred and thirty-six tyrosine phosphorylation sites were then quantified and VPS34-IN1 was found to decrease the phosphorylation of 11 of these by at least 45% (Fig. 5C). The 11 proteins whose phosphorylation on tyrosine was inhibited by VPS34-IN1 in MOLM-14 cells are listed in Supplementary Table 4 and Excel file S1. These factors included STAT5 and, to a lesser extent, MAPK1/3. These proteins are key actors downstream of FLT3-internal tandem duplication (ITD) signaling in AML. Interestingly, MOLM-14 and MV4-11 cell lines, which harbor FLT3-ITD mutations, were the most sensitive to the antileukemic activity of VPS34-IN1, PIK-III, or Autophinib in our current experiments (Fig. 1A and Supplemental Figs. 2A, 3B). To confirm this increased sensitivity in FLT3 mutant AML cells to VPS34 inhibitors, we used BA/F3 cells which are IL-3-dependent for growth and survival. BA/F3 cells engineered to express the FLT3-ITD mutations showed an increased sensitivity to VPS34 inhibition compared to BA/F3 wild type (Fig. 5D and Supplemental Fig. 7). Interestingly, using data from Beat AML project^36^, we observed that the expression of VPS34 was significantly increased in FLT3 mutant AML (Fig. 5E). Altogether, these data suggest an elective VPS34 dependency in this sub-type of AML, and a role of VPS34 in FLT3 mutant signaling.

We next tried to confirm the results of phosphoproteomic analysis. We observed that VPS34-IN1 strongly inhibited STAT5 phosphorylation in MOLM-14 and in MV4-11 cells lines, whereas it had no effect on STAT5 phosphorylation in the U937 and K562 cell lines that do not contain the FLT3-ITD mutation (Fig. 5F and Supplemental Fig. 8). Autophinib and PIK-III were also able to inhibit STAT5 phosphorylation in MOLM-14 and in MV4-11 cell lines, which suggest that this effect was specific to VPS34 inhibition (Supplemental Fig. 4C). Strikingly, VPS34-IN1 had a lesser impact on the phosphorylation of P42/44MAPK, suggesting that it mostly acts on the hypoglycosylated form of FLT3-ITD which activates STAT5 at the endoplasmic reticulum (Fig. 5F). VPS34-IN1 had no effect on STAT5 or P42/44 MAPK phosphorylation in wild type BA/F3 cells cultured with IL-3. In contrast, BA/F3 cells engineered to express the FLT3-ITD mutations were sensitive to VPS34-IN1 in term of STAT5 and P42/44 MAPK phosphorylation inhibition (Fig. 5G). We determined the phosphorylation status of the FLT3 receptor in MOLM-14 and BA/F3 FLT3-ITD cells treated or not with a short exposure to VPS34-IN1. FLT3 receptor phosphorylation was inhibited in the presence of the specific FLT3 tyrosine kinase inhibitor AC220, but not in presence of VPS34-IN1 (Fig. 5H). These results collectively demonstrated that VPS34-IN1 inhibits STAT5 phosphorylation in AML cells expressing FLT3 mutations. This effect was not related to an inhibition of the kinase activity of FLT3 but probably by blocking the downstream signal transmission on STAT5. They further indicate that VPS34 controls the tyrosine phosphorylation of a restricted set of signaling proteins that have been implicated in AML biology.

In clinical practice, a recurrent phenomenon in patients receiving FLT3 inhibitors is the emergence of leukemia clones carrying FLT3-TKD (tyrosine kinase domain) mutations at relapse^37^. Mutations may occur at the activation loop (D835V) and confer resistance to AC220 and Sorafenib or at gate-keeper site (F691L), which also confer partial resistance to crenolanib^38^. As expected, concomitant FLT3-ITD and FLT3-TKD mutations confers resistance to AC220 and sorafenib and mildly reduced sensitivity to crenolanib (Fig. 5I). Interestingly, we showed that VPS34 inhibitors were still able to inhibit STAT5 phosphorylation and to impair cell viability at the same level in BA/F3 cells engineered with concomitant FLT3-ITD and FLT3-TKD mutations (Fig. 5G–I and Supplemental Fig. 4).

## Discussion

We here report that the inhibition of class III PI3K using the VPS34-IN1 inhibitor has antileukemic activity in a large panel of AML cell lines and in most primary AML cells. We demonstrate that VPS34-IN1 causes pleiotropic effects on various cellular functions related to class III PI3K in AML cells, and that this may explain their survival impairment upon exposure to this inhibitor (Supplemental Fig. 4).

We first analyzed the ability of VPS34-IN1 to inhibit the canonical autophagy pathway in which VPS34 exerts a pivotal role. Cancer cells are thought to exhibit high autophagy flux to support their rapid proliferation and turnover rates. Our analysis indicated that VPS34-IN1 inhibits the basal autophagy flux in AML cells. Prior data regarding the basal autophagy flux and the consequence of its inhibition in AML cells are conflicting, with some studies suggesting its role in cell survival and proliferation and others not^7,18,39–41^. This discrepancy may be explained both by methodological variabilities between studies and by the inherently heterogeneous nature of AML, especially at the molecular level. Similarly, autophagy may also represent a protective or a contributing mechanism to cell death mediated by different AML treatments^13–16^. Given the ability of VPS34-IN1 to strongly and rapidly inhibit basal autophagy, we tested its activity in a model of AML therapy which induces strong autophagy. In a previous study, we demonstrated the antileukemic activity of l-asparaginase in AML that mimics extracellular glutamine depletion leading to mTORC1 inhibition, OXPHOS inhibition and mitochondrial apoptosis^11^. Preliminary results suggested that this compound induces autophagy. In our present study, we confirmed that l-asparaginase induces strong autophagy in AML cells and that this was blocked by VPS34-IN1. However, VPS34-IN1 synergistic proapoptotic effects with l-asparaginase in AML cells suggested that the inhibition of l-asparaginase-induced autophagy may help to potentiate its antileukemic activity. Thus, the combination of VPS34-IN1 with treatments that promote autophagic mechanisms of resistance to cell death could represent a new therapeutic strategy for AML.

Beyond its key role in autophagy, class III PI3K is a major factor in vesicular trafficking. Hence, VPS34-IN1 causes the accumulation of intracellular late endosomes, lysosomes and lipid droplets, as described previously in VPS34 KO experiments^27,34,42^. This led us to study the impact of endocytic trafficking impairment on the intracellular signaling of AML. Lysosomes and late endosomes play a central role in the signaling from mTORC1 as they serve as signaling platforms for this molecule. We showed in our present experiments that class III PI3K inhibition leads to the inhibition of mTORC1 signaling, one of the principal signaling pathways in AML^35^. The relationship between mTORC1 and VPS34 is paradoxical. On one hand, mTORC1 phosphorylates ULK1 and inhibits VPS34 ULK1-dependent activation. On the other hand, VPS34 produces PI3P, allowing recruitment of the PI3P-binding protein FYCO1 to lysosomes and promoting contacts between FYCO1 on lysosomes and the endoplasmic reticulum that contains the PI3P effector protrudin. This brings the lysosomal mTORC1 complex in close proximity to nutrient signaling complexes at the plasma membrane and increases mTORC1 activity^43^. AMPK is another critical sensor of the intracellular energy level and a fine-tuner of cell metabolism^44^. A recent study has reported that VPS34 inhibition alters the cellular energy metabolism and lowers the intracellular ATP levels^29^. This leads to the activation of the AMPK pathway in liver and muscle. Thus, by modulating both the mTORC1 and AMPK pathways, class III PI3K may emerge as a major mediator of metabolic homeostasis and of the viability of AML cells. Further studies on the metabolic consequences of class III PI3K inhibition will be needed to provide a comprehensive understanding of the role of this kinase on the metabolic control in AML.

We also investigated whether the inhibition of class III PI3K in AML cells could impact other signaling pathways. Using an unbiased phosphoproteomic approach, we identified FLT3 mutant signaling as a new pathway controlled by class III PI3K. FLT3 mutations are found in 30% of newly diagnosed AML and arise essentially as internal tandem duplications (FLT3-ITD) or less frequently as Tyrosine domain mutations (FLT3-TKD). FLT3-ITD mutations constitutively activate the FLT3 kinase, resulting in the proliferation and survival of AML cells. Mislocalized premature activation of FLT3-ITD at intracellular locations alters the use of signaling pathways and represents a cause of the aberrant activation of signaling by this mutant receptor^45^. FLT3-ITD is efficiently phosphorylated intracellularly, but only on specific tyrosines such as Y591. Further trafficking of the mutant receptor to the plasma membrane then results in phosphorylation of different tyrosines, such as Y842. Phosphorylation of FLT3-ITD at the plasma membrane initiates a different spectrum of signaling events, for example the PI3K and ERK/MAPK pathways. Phosphorylation of FLT3-ITD at the endoplasmic reticulum controls STAT5 signaling^45,46^. Here, we show that MOLM-14 and MV4-11 cell lines, which harbor FLT3-ITD mutations, were the most sensitive to the anti-leukemic effects of VPS34-IN1 and other VPS34 inhibitors. BA/F3 cells engineered to express the FLT3-ITD mutations also showed an increased sensitivity to VPS34 inhibition compared to BA/F3 wild type. In these cells, VPS34-IN1 also induced the dose-dependent inhibition of constitutive STAT5 phosphorylation and a downregulation of the MAPK signaling pathway. FLT3 receptor phosphorylation was not inhibited by VPS34-IN1, in contrast to classical FLT3 tyrosine kinase inhibitor. This suggests that the inhibition of FLT3 mutant signaling that we observed is not related to an off-target effect of VPS34-IN1 but rather to a specific role of class III PI3K downstream of FLT3-ITD mutant signaling. It could be hypothesized that VPS34-IN1, through the impairment of the vesicular trafficking, alters the localization of the FLT3 mutant to the plasma membrane or the endoplasmic reticulum leading to FLT3 mutant signaling inhibition. Another hypothesis is that the production of PI3P by PI3K class III on the surface of the endoplasmic reticulum is essential for the contact between mutant FLT3 and STAT5 via an unknown scaffold protein carrying a PX or FYVE domain. We also observed STAT5 inhibition in a model of FLT3-ITD/FLT3-TKD co-mutation, which represents a mechanism of acquired resistance to specific FLT3-ITD inhibitors in vivo^47^. Interestingly, the antileukemic activity of VPS34 inhibitor was maintained in this comutation model. An understanding of the mechanisms underlying these observations is essential. Indeed, this could represent a new therapeutic strategy in this AML subtype.

One of the issues to consider regarding the systemic use of VPS34 inhibitors as anticancer drugs is the possibility of serious side effects^34^. Many studies have reported that the complete ablation of VPS34 leads to profound cellular and organ damage that could limit the therapeutic use of these treatments^34,48,49^. We here observed however that VPS34-IN1 does not alter the viability of normal immature CD34+ hematopoietic cells, suggesting a potential therapeutic window for its use in AML. Only one VPS34 inhibitor has been thus far tested in vivo, which is compound 19, a structural analog of VPS34-IN1^50^. In that proof-of-concept study, compound 19 was tested over a 5-week period at a relatively low dose of 20 mg/kg^29^. This treatment regimen was well-tolerated and showed improved glucose tolerance in a mice model of type 2 diabetes, suggesting that systemic treatments with VPS34 inhibitors may be feasible. A major drawback of compound 19 however is its very short in vivo half-life, which has limited its use in cancer models for instance^50^.

Finally, our present findings indicate that class III PI3K is a major kinase in AML cell biology and controls autophagy, vesicular trafficking and major signaling pathways, and highlight the potential for targeting class III PI3K as a strategy to treat AML (see Supplemental Fig. 9 for a schematic representation of the role of class III PI3K of in AML).

## Supplementary information

Supplementary legend and material

Supplemental Table 1

Supplemental table 2

Supplemental Table 3

Supplemental table 4

Supplemental Figure 1

Supplemental Figure 2

Supplemental Figure 3

Supplemental Figure 4

Supplemental Figure 5

Supplemental Figure 6

Supplemental Figure 7

Supplemental Figure 8

Supplemental Figure 9
