# Supplementary material for: Antileukemic activity of the VPS34-IN1 inhibitor in acute myeloid leukemia
Source: Oncogenesis. 2020 Oct 22;9(10):94. doi: 10.1038/s41389-020-00278-8 (PMC7581748; doi:10.1038/s41389-020-00278-8)
Supplement: Supplementary file 1 — Supplementary legend and material [file 41389_2020_278_MOESM1_ESM.docx]

**Supplemental figure legends**

***Supplemental Figure 1:* A/** MOLM-14 scrambled (SCR) cells or MOLM-14 shVPS34 cells were cultured with or without L-asparaginase (10 UI/ml) and or hydroxychloroquine (10 µM), as indicated. VPS34, LC3-I and LC3-II expression were then assessed by western blot. **B/** VSP34 expression in MOLM-14 SCR cells and in MOLM-14 cells in which VPS34 expression was disrupted using CRISPR/CAS9 technology (subclones) was assessed by western blot.

***Supplemental Figure 2:* A/** AML cell lines were cultured for 48 hours in the presence of PIK-III or Autophinib over a large concentration range and viability was quantified using the fluorescence-based Uptiblue assay. This enabled a determination of the IC_50_ values for PIK-III and autophinib; n=3, error bars represent the standard deviation. **B/** AML cell lines were cultured for 48 hours with vehicle or 1, 3, 10, 30 or 100µM of PIK—III or Autophinib. Cell death was quantified by flow cytometry analysis of the percentage of annexin-V positive cells; n=3, bars represent the standard error of the mean.

***Supplemental Figure 3:* A/** Western blot analysis was used to evaluate the amount of VPS34 in AML cell lines from figure 1-A. **B/** Correlation between IC50 and the ratio of VPS34/actin was performed using Spearman correction. Simple linear regression with CI 95% is also represented. **C/** Correlation between relative VPS34 expression from Proteomics^57^ (DepMap Portal) and VPS34 gene effect (CERES) from CRISPR (Avana) public^58^ (DepMap Portal) was performed using Spearman correction. Simple linear regression with CI 95% is also represented. The CERES dependency score is based on data from a cell depletion assay. A lower CERES score indicates a higher likelihood that the gene of interest is essential in each cell. A score of 0 indicate a gene is not essential; correspondingly -1 is comparable to the median of all pan-essential genes.

***Supplemental Figure 4:* A/** MOLM-14 cells were cultured for 24 hours with vehicle or PIK-III or Autophinib (10 µM) and observed by optical microscopy (X100) after May Gruenwald Giemsa staining. **B/** MOLM-14 cells were cultured for 24 hours with vehicle (left panel) or PIK-III or Autophinib (10 µM) and MFI was measured following Lysotracker deep red staining.**C/** MOLM-14 and MV4-11 cells were cultured for 6 hours with vehicle or chloroquine (10 µM) and 10 µM of PIK-III or Autophinib. Western blot analysis was then performed using antibodies directed against pSTAT5, STAT5, LC3-II and actin. **D/** MOLM-14 and MV4-11 cells were cultured for 24 hours with vehicle or PIK-III (10 µM) and p62 accumulation was assessed by western blotting.

***Supplemental Figure 5:* A/** MOLM-14 cells were cultured with L-asparaginase at 10 UI/L for 1, 4, 6 or 24 hours. Autophagy induction was assessed by analyzing LC3-I and LC3-II expression by western blot **B/** The mean fluorescence intensity quantification of GFP with and without saponin in vehicle- or L-asparaginase-treated (10UI/ml) MOLM-14 cells was determined using flow cytometry; n=3, bars represent the standard error of the mean. **C/** Primary AML samples from 4 patients were cultured for 24 hours with L-asparaginase (10 UI/ml) and or chloroquine (10 µM) as indicated and autophagy induction was evaluated by western blotting analysis of LC3-I and LC3-II expression. **D/** MOLM-14 cells or primary AML samples were cultured for 48 hours with vehicle or L-asparaginase (10 UI/ml) alone, VPS34-IN1 (5µM) alone or L-asparaginase + VPS34-IN1. Apoptosis was then quantified by annexin-V staining; n=3, bars represent the standard error of the mean.

***Supplemental Figure 6:*** Synergy map (left panel) and viability matrix (right panel) of primary AML samples cultured for 48h in the presence of L-asparaginase and VPS34-IN1.

***Supplemental Figure 7:*** BA/F3 WT and BA/F3 cells with FLT3-ITD expression, with or without IL-3, were cultured for 48 hours in the presence of PIK-III or Autophinib over a large concentration range. Viability was quantified using the fluorescence based Uptiblue assay; n=3, bars represent standard error of the mean.

***Supplemental Figure 8:*** MOLM-14 cells were cultured for 6 hours with vehicle or increasing concentrations of VPS34-IN1. Western blot analysis was used to study the activation of the STAT5 pathway using an anti-pSTAT5 antibody and of the mTORC1 pathway using an anti-Thr 389 pP70S6K antibody.

***Supplemental Figure 9. Schematic representation of the role the class III PI3K of in AML cells.*** (a) In AML cells, the class III PI3K (VPS34-VPS15) acts with other proteins to form a specialized complex involved in vesicular trafficking, autophagy initiation and autophagosome maturation. VPS34 is also involved in the control of various cell signaling pathways, including mTORC1 and FLT3-ITD via still unknown mechanisms. (b) During VPS34-IN1 treatment, the acute and complete inhibition of the VPS34 kinase domain leads to abrogation of the cellular functions controlled by VPS34, to mTORC1 inhibition and specifically to STAT5 inhibition downstream of FLT3-ITD, resulting in acute cell death.

**Supplemental Materials and Methods.**

**Lentivirus production and AML cell line infection:** 293-T packaging cells were used to produce constructs through the co-transfection of these cells with plasmids encoding lentiviral proteins. Supernatants were collected and ultracentrifuged for 48 hours after transfection over two consecutive days, and then stored at -80°C. AML cell lines were plated at 2x10^6^/ml and 2-10μl aliquots of lentiviral supernatants were added for 3 hours. Cells were then grown in 10% FCS medium and further selected with puromycin or cell sorted with an ARIA 3 cytometer. In experiments using inducible shRNAs, 200μg/ml doxycycline was added to the culture medium.

**Phosphoproteomic analysis**

MOLM-14 cells were cultivated for 2 weeks with heavy lysine (+8 Da) and heavy arginine (+10 Da). At the end of the culture period, more than 90% of Arg and Lys in the proteins were of the heavy forms. Two experiments were then performed. In the first of these, heavy cells were treated with 5µM VPS34-IN1 for 1 hour while light cells received vehicle only (DMSO). At the end of the experiment, cells were recovered and the heavy (VPS34-IN1-treated) and light (control) cells were mixed. In the second experiment, light cells were treated with VPS34-IN1 and heavy cells were used as a control. After PBS washing, 50x10^6^ mixed cells were incubated for 1 hour at room temperature with 1 ml buffer A (100 mM Tris/HCl pH8.00 containing 8M urea, 1 mM sodium glycerophosphate, 2.5 mM sodium pyrophosphate, 1 mM sodium vanadate, 20 mM tris (2-carboxyethyl)phosphine (TCEP) and 50 mM sodium chloroacetamide). The cell extract was then diluted with 3 ml 100 mM Tris/HCL pH 8.00 and incubated overnight with 400 µg trypsin. Peptides were cleaned by chromatography through SepPak columns and dried. Two mg of peptides were used for phosphopeptide purification using TiO2 (Titansphere, GL Science) and 2 mg were also used for the purification of phosphotyrosine-containing peptides using anti-phosphotyrosine antibodies. Two successive immunoprecipitations were performed using PY100 antibodies in the first instance (Cell Signaling Technology) and then protein-sepharose (GE Healthcare; Chicago, IL), followed by a mixture of agarose-bound PY99 (Santa-Cruz; Heidelberg, Germany) and 4G10 (Millipore; Darmstat, Germany) antibodies.

Mass spectrometry analyses were performed using a Dionex U3000 RSLC nano-LC system coupled to an Orbitrap Fusion mass spectrometer (Thermo Fisher Scientific). Peptides were solubilized in 0.1% trifluoracetic acid (TFA) containing 10% acetonitrile (ACN) and separated on a C18 column (2 µm particle size, 75 µm inner diameter, 15 cm length; Thermo Fisher Scientific) with a 3 hour gradient (TiO2 purified peptides) or a 1 hour gradient (anti-phosphotyrosine purified peptides) starting from 99% solvent A (0.1% formic acid) and ending with 55% solvent B (80% ACN, 0.085% formic acid). The mass spectrometer was operated in a data-dependent manner and full MS scans were acquired with the Orbitrap, followed by HCD fragmentation of the most abundant ions and detection in the ion trap for 3 seconds.
