# Supplementary material for: Antileukemic activity of the VPS34-IN1 inhibitor in acute myeloid leukemia
Source: Oncogenesis. 2020 Oct 22;9(10):94. doi: 10.1038/s41389-020-00278-8 (PMC7581748; doi:10.1038/s41389-020-00278-8)
Supplement: Supplementary file 2 — Supplemental Table 1 [file 41389_2020_278_MOESM2_ESM.pdf]

	Age	AML type	BM vs PB	WBC (G/L)	Blast (%)	Cytogenetics	FLT3 ratio	Molecular status	% of annexin V + cells without VPS34-IN1	% of annexin V + cells with VPS34-IN1	Delta of % annexin V+ cells
AML1	88	post MDS		17	50	del7	-	N/D	29	41	12
AML2	69	AML2	PB	0,6	20%	del7	-	IDH2, FLT3-	24	54	30
AML3	52	AML5	PB	17,7	37%	Del7 Tri8	-	FLT3-, NPM1-, IDH1/2-	31	44	13
AML4	84	post MDS	BM	1,24	74%	del7	-	FLT3-, NPM1-, IDH1/2-	30	42	12
AML5	66	N/D	BM	2,21	80	Complex	-	FLT3-, NPM1-, IDH1/2-	32	52	20
AML6	24	AML4	BM	78,2	38	inv16	-	FLT3-, NPM1-, IDH1/2-	8	25	17
AML7	79	N/D	PB	60,6	80%	N/D	-	N/D	8	13	5
AML8	77	post-CMML	PB	120	>80	Normal	-	SRSF2+	18	19	1
AML9	90	N/D	PB	80	>80	N/D	-	N/D	22	37	15
AML10	73	AML0	PB	12,4	87	Normal	-	AML1+, RAS+	19	25	6
AML11	55	AML4	PB	74,7	88%	Normal	1,08	NPM1+ FLT3-ITD+	8	22	14
AML12	63	AML 0	BM	50	70%	Complex	-	NPM1+, DNMT3A+, NRAS+	25	35	10
AML13	74	N/D	BM	0,3	82%	Normal	-	IDH2 R172K	23	58	35
AML14	67	post-MDS	PB	1,74	64	Complex	-	EVI1	7	66	59
AML15	82	post-MDS	PB	59	60	N/D	-	N/D	13	12	-1
AML16	53	AML6	PB	53	98	t(2,18)	-	FLT3-, NPM1-, IDH1/2-	5	32	27
AML16R	-	-	PB	40	83	-	-	-	39	63	24
AML17	43	N/D	BM	0,79	77%	Normal	-	IDH2 R172K	18	48	30
AML18	74	post-MDS	BM	3,2	66%	Normal	0,05	NPM1+, FLT3-ITD+, IDH2 R172K, CEBPA+	34	62	28
AML19	58	N/D	BM	295	68%	Normal	-	Nras+, EZH2+	40	81	41
AMLR19	-	-	BM	-	-	Normal	-	-	45	59	14
AML20	71	post-ET	BM	23	92%	Normal	-	IDH1 R132C, FLT3 ITD-	65	92	27
AML21	56	N/D	BM	100	87%	Normal	-	NPM1+, FLT3 ITD-	16	31	15
AML22	35	N/D	BM	50	97%	Normal	11,6	FLT3-ITD+, STAG2 +	N/D	N/D	N/D
AML23	68	post-MDS	PB	77,5	95%	inv 16	-	CBFb-MYHB+, BCOR+, KIT+, TET1+, FLT3-TKD+	N/D	N/D	N/D
AML24	85	N/D	BM	N/D	84%	N/D	-	FLT3-, NPM1-, IDH1/2-	N/D	N/D	N/D
AML25	53	N/D	PB	N/D	100%	N/D	0,69	FLT3-ITD+	N/D	N/D	N/D
AML26	32	AML2	PB	215	28%	Normal	0,069	WT1+, FLT3 ITD+	N/D	N/D	N/D
AML27	56	N/D	BM	N/D	100%	Complex	-	IDH2+, TP53+, STAG2+, PHF6+, ASXL1+	N/D	N/D	N/D

Supplemental Table 1
