# Supplementary material for: Antileukemic activity of the VPS34-IN1 inhibitor in acute myeloid leukemia
Source: Oncogenesis. 2020 Oct 22;9(10):94. doi: 10.1038/s41389-020-00278-8 (PMC7581748; doi:10.1038/s41389-020-00278-8)
Supplement: Supplementary file 3 — Supplemental table 2 [file 41389_2020_278_MOESM3_ESM.pdf]

Name	FAB	Cytogenetics	Molecular	References
MOLM 14	AML4 post MDS	MLL-AF9	FLT3-ITD1	Matsuo Y (leukemia 1997)
THP1	AML5 de novo	MLL-AF9	N-ras, p53	Odero MD (genes chromosomes and cancer 2000)
MV4-11	biphenotypic	Complex	FLT3-ITD (homozygous)	Lange (Blood 1987)
HL-60	AML2	MYC amplification	N-ras, CCND2 C238T4, p53 homozygous deletion	Ikediobi ON (mol cancer ther 2006),Collins (Nature 1977)
U937	AML5	t(10;11)(p14;q23)	p53	Sunstrom C (int j cancer 1976)
KASUMI	AML2	t(8;21)	c-kit N822K	Asou H (Blood 1991); Larizza l (Leuk lymph 2005)
OCI-AML2	AML4	N/D	DNMT3A	Tiacci A (leukemia 2012)
OCI-AML3	AML4	N/D	DNMT3A, NPM1	Wang (Leukemia 1989)
K562	blast crisis CML	N/D	p53, Bcr-Abl	Lozzio (J Natl Cancer Inst 1973)

Supplemental Table 2
