# Supplementary material for: Antileukemic activity of the VPS34-IN1 inhibitor in acute myeloid leukemia
Source: Oncogenesis. 2020 Oct 22;9(10):94. doi: 10.1038/s41389-020-00278-8 (PMC7581748; doi:10.1038/s41389-020-00278-8)
Supplement: Supplementary file 4 — Supplemental Table 3 [file 41389_2020_278_MOESM4_ESM.pdf]

Antibody	Manufacturer	Reference
Anti phospho p70S6K (T389)	Cell signaling	9205
Anti phospho 4EBP1 (S65)	Cell signaling	9451
Anti phospho ULK (S757)	Cell signaling	6888
Anti phospo STAT5	Cell signaling	93515
Anti phospho FLT3 Y591	Cell signaling	3474
Anti phoshpo AKT T308	Cell signaling	13038
Anti phospho AKT S473	Cell signaling	9260
Anti p42/p44 MAPK	Cell signaling	4377
Anti p70S6K	Cell signaling	2708
Anti LC3B	Cell signaling	2775
Antiβ-actin	Sigma	A-74
Anti ULK	Cell signaling	4473
Anti STAT5	Santa Cruz technologies	sc-74442
Anti FLT3	Cell signaling	3462
Anti caspase 3	Cell signaling	9665
Anti AKT	Cell signaling	9272
Anti PARP	Cell signaling	9542
Anti 4EBP1	Cell signaling	9452
Anti Caspase 3	Cell signaling	9662
Anti Cleaved caspase 3	Cell signaling	9664
Anti Caspase 8	Cell signaling	9746
Anti VPS34	Cell signaling	4263
Anti ATG14	Cell signaling	5045
Anti UVRAG	Cell signaling	5320
Anti Beclin1	Santa Cruz technologies	sc-48341
Anti Rubicon	Cell signaling	7151
Anti VPS15	Proteintech	17894-1-AP
Anti p85	Raised in house	-
Anti STAT5	Santa Cruz technologies	SC 835

Supplemental Table 3
