# Supplementary material for: Antileukemic activity of the VPS34-IN1 inhibitor in acute myeloid leukemia
Source: Oncogenesis. 2020 Oct 22;9(10):94. doi: 10.1038/s41389-020-00278-8 (PMC7581748; doi:10.1038/s41389-020-00278-8)
Supplement: Supplementary file 5 — Supplemental table 4 [file 41389_2020_278_MOESM5_ESM.pdf]

Protein	Gene	Position PhoshoTyr	% Inhibition of phosphoTyr with VPS34 IN1
Signal transducer and activator of transcription	STAT5A STAT5B	694; 664; 182; 699	82
Tyrosine-protein kinase HCK	HCK	188; 209	66,5
Phosphatidylinositol3,4,5-triphosphate 5-phosphatase 2 SHIP2	INPPL1	921; 745; 987	62,3
Calcium-binding protein 39	CAB39	323;325	58,1
ATP synthase subunit, mitochondrial	ATP5H	126; 150	54,5
Protein jagged-1	JAG1	980; 1139	54,1
Mitogen-activated protein kinase 1	MAPK1	187	51,7
Annexin A2, Putative annexin A2-like, Annexin	ANXA2, ANXAP2	24; 42; 52	50,9
Actin	ACTB, ACTG1	198; 189	48,9
Tyrosine-protein kinase Lyn	LYN	295; 316	46,8
Mitogen-activated protein kinase 3	MAPK3	90; 204	45,9

Supplemental Table 4
