# Supplementary figures and images for: Antileukemic activity of the VPS34-IN1 inhibitor in acute myeloid leukemia
Source: Oncogenesis. 2020 Oct 22;9(10):94. doi: 10.1038/s41389-020-00278-8 (PMC7581748; doi:10.1038/s41389-020-00278-8)

A

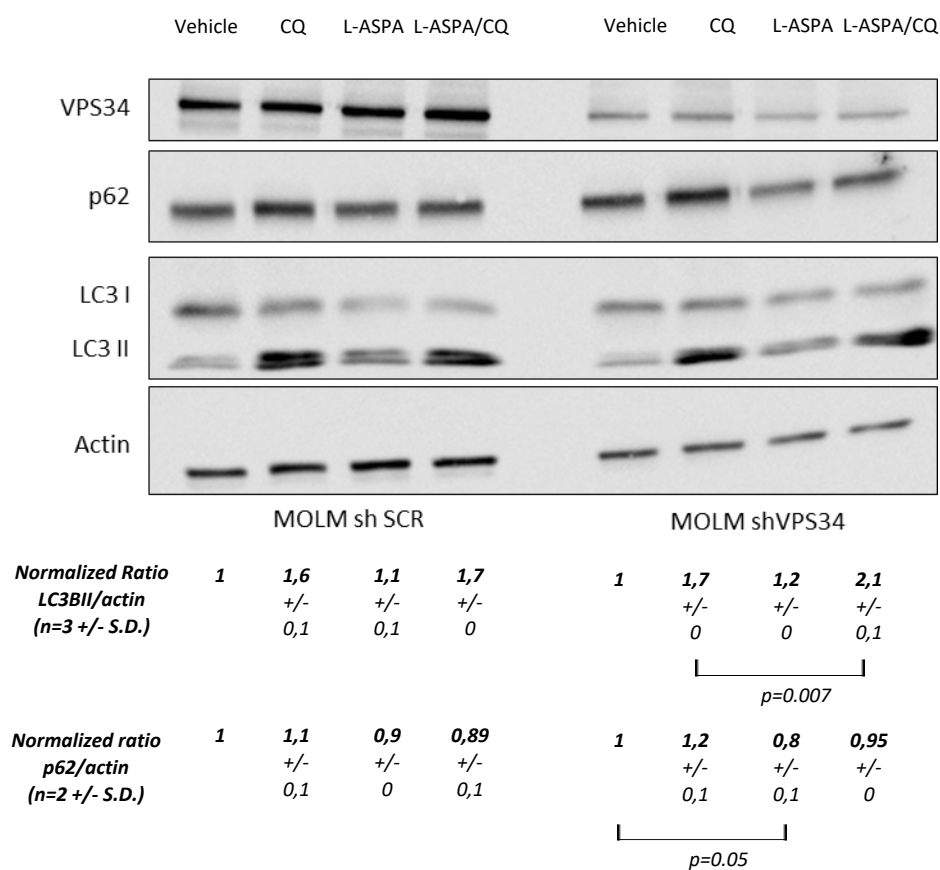

B

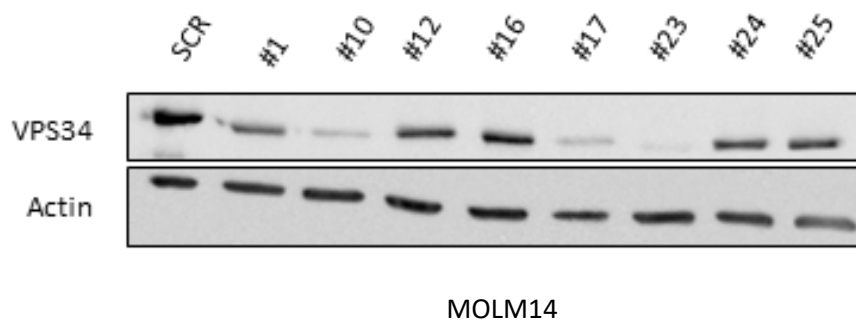

Supplement: Supplementary file 6 — Supplemental Figure 1 [file 41389_2020_278_MOESM6_ESM.pdf]

A

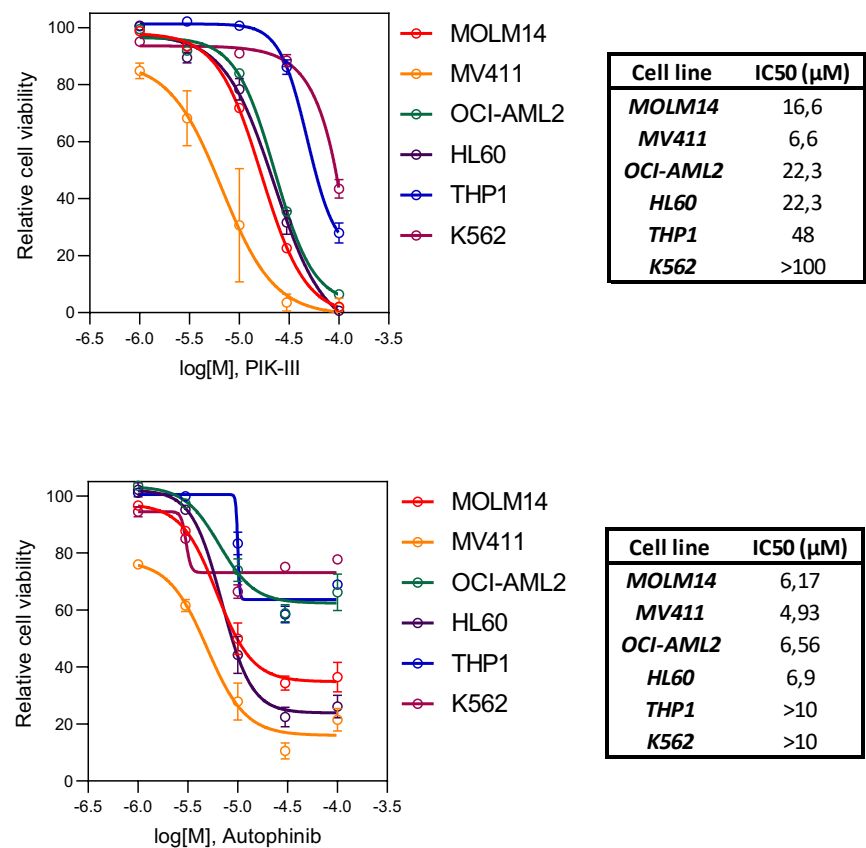

B

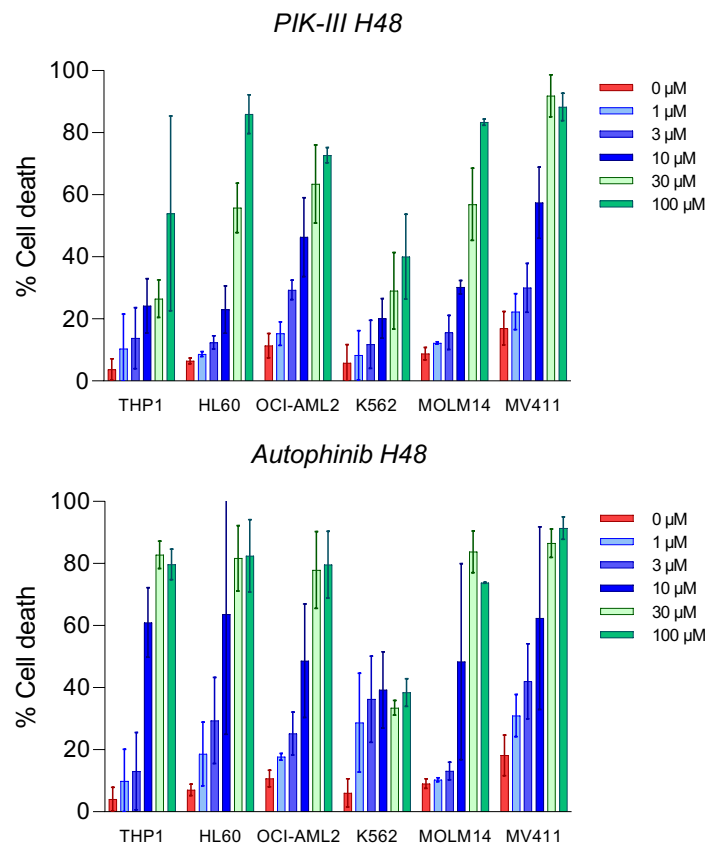

Supplement: Supplementary file 7 — Supplemental Figure 2 [file 41389_2020_278_MOESM7_ESM.pdf]

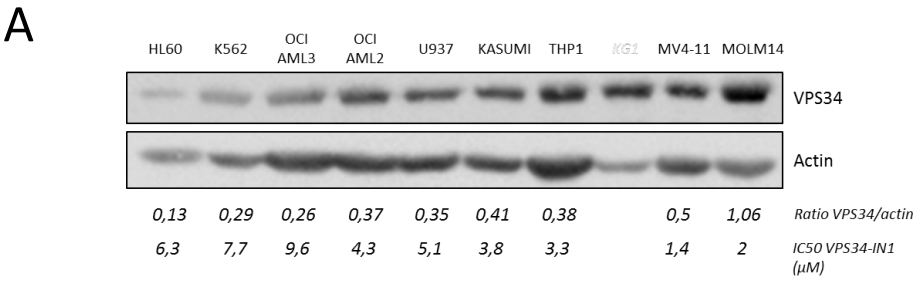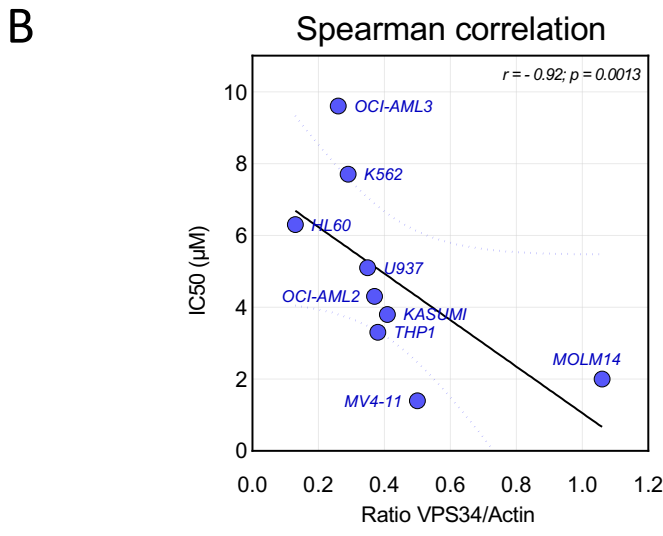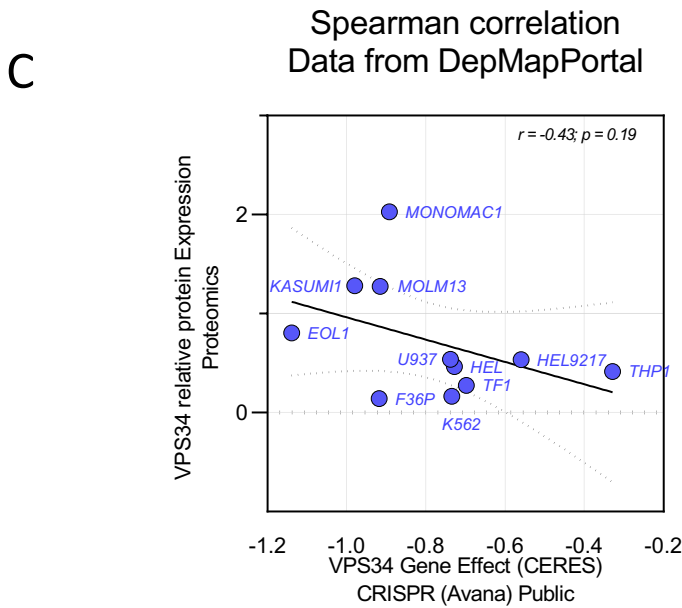

Supplemental Figure 3

Supplement: Supplementary file 8 — Supplemental Figure 3 [file 41389_2020_278_MOESM8_ESM.pdf]

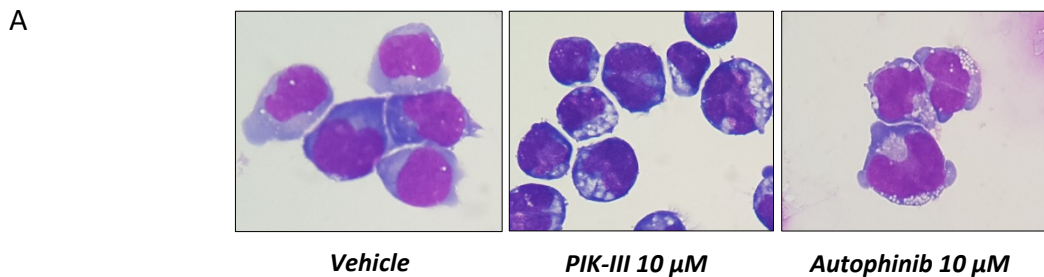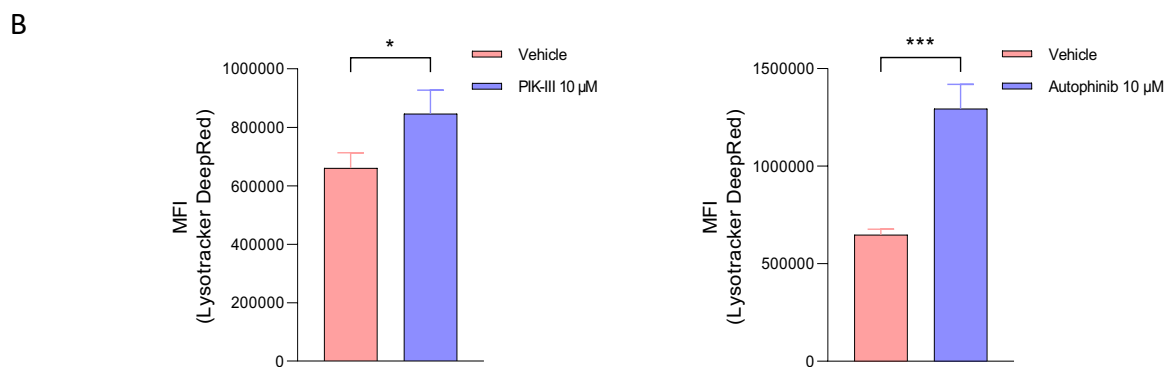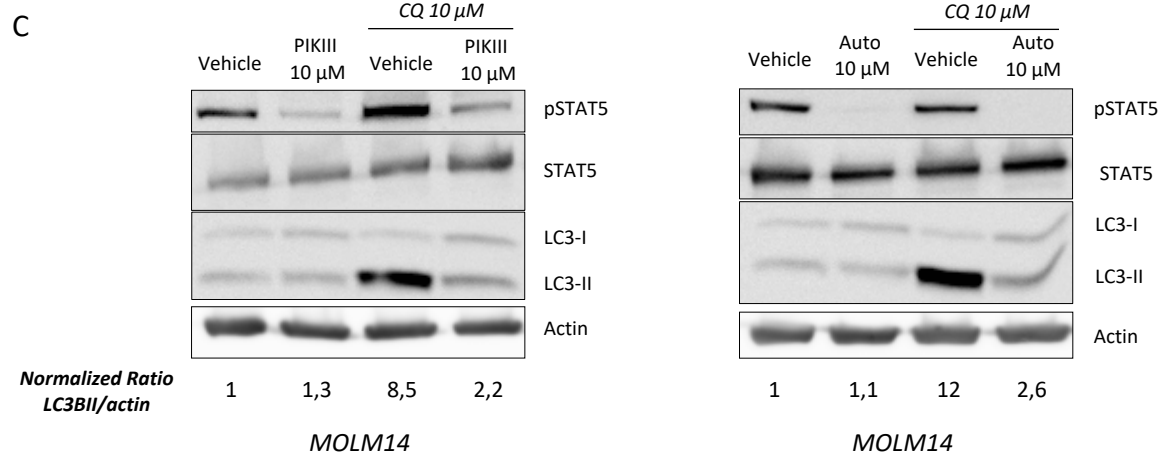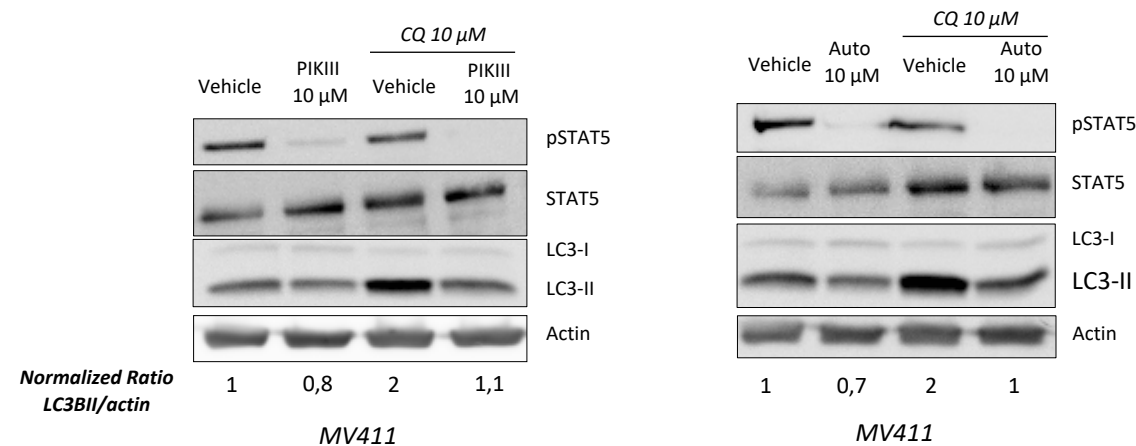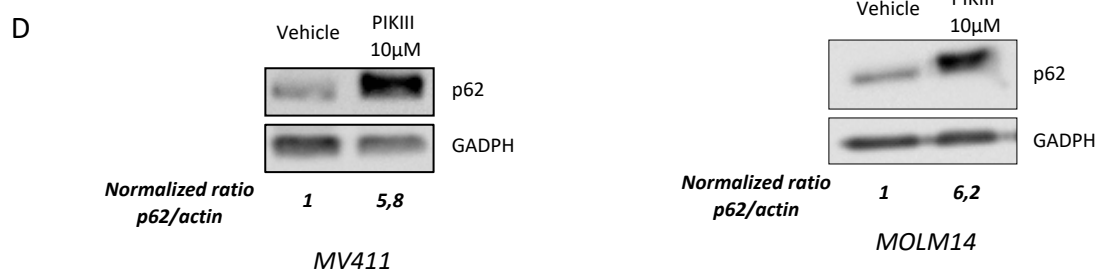

Supplemental Figure 4

Supplement: Supplementary file 9 — Supplemental Figure 4 [file 41389_2020_278_MOESM9_ESM.pdf]

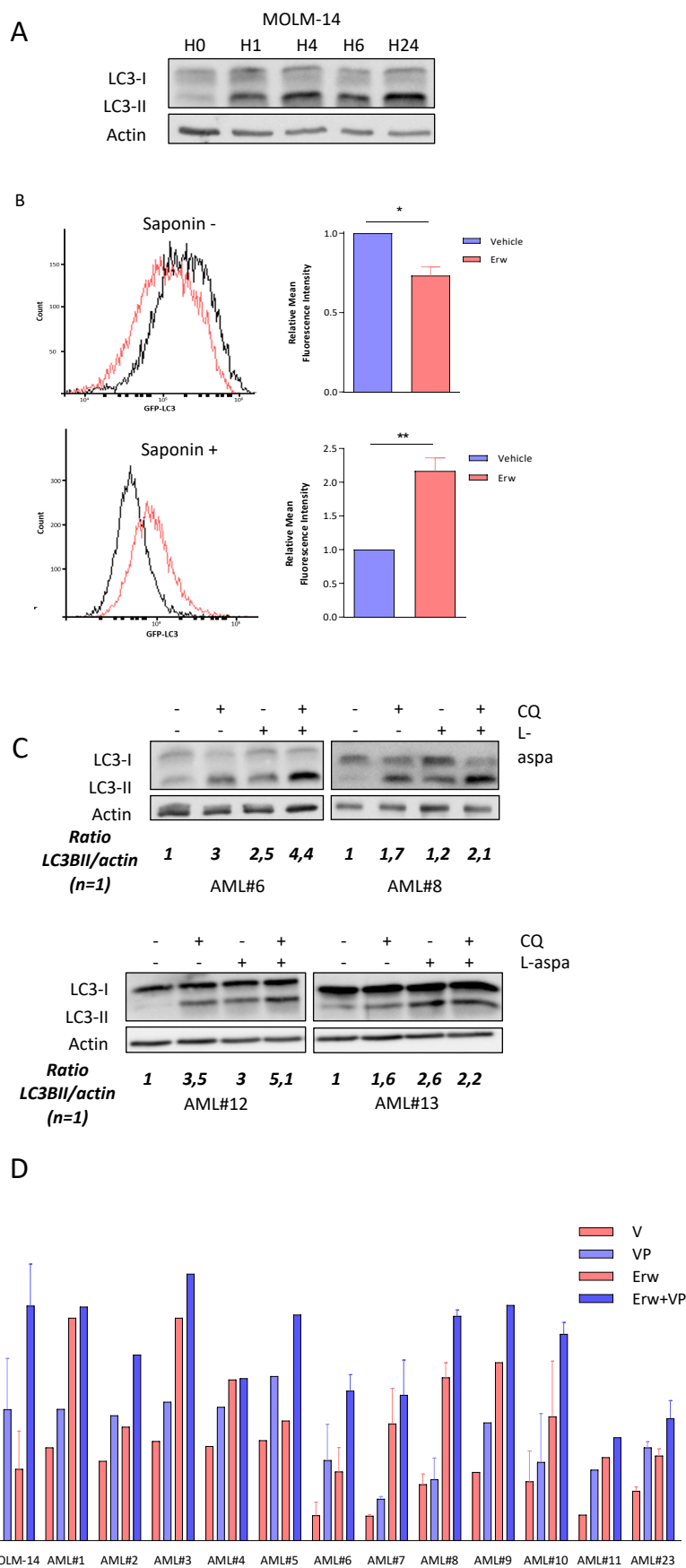

Supplemental Figure 5

Supplement: Supplementary file 10 — Supplemental Figure 5 [file 41389_2020_278_MOESM10_ESM.pdf]

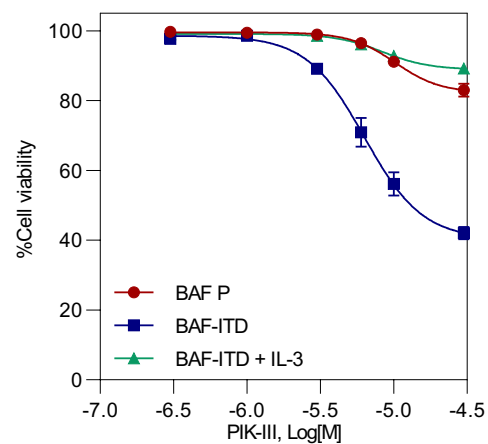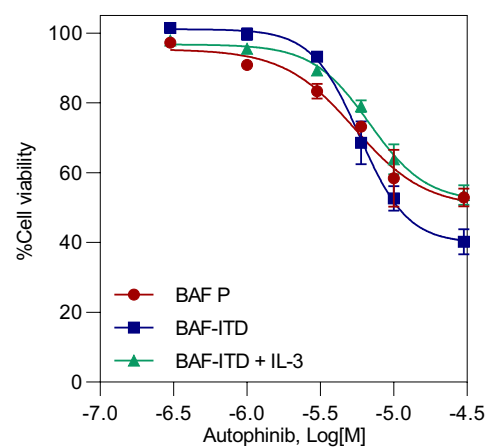

Supplemental Figure 7

Supplement: Supplementary file 12 — Supplemental Figure 7 [file 41389_2020_278_MOESM12_ESM.pdf]

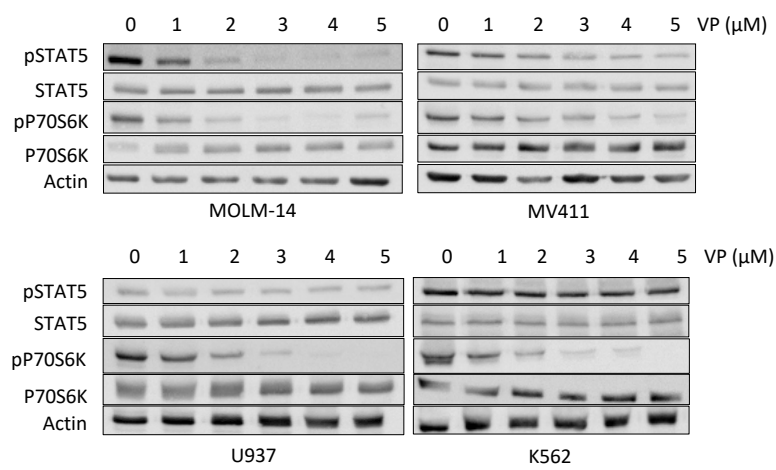

Supplemental Figure 8

Supplement: Supplementary file 13 — Supplemental Figure 8 [file 41389_2020_278_MOESM13_ESM.pdf]

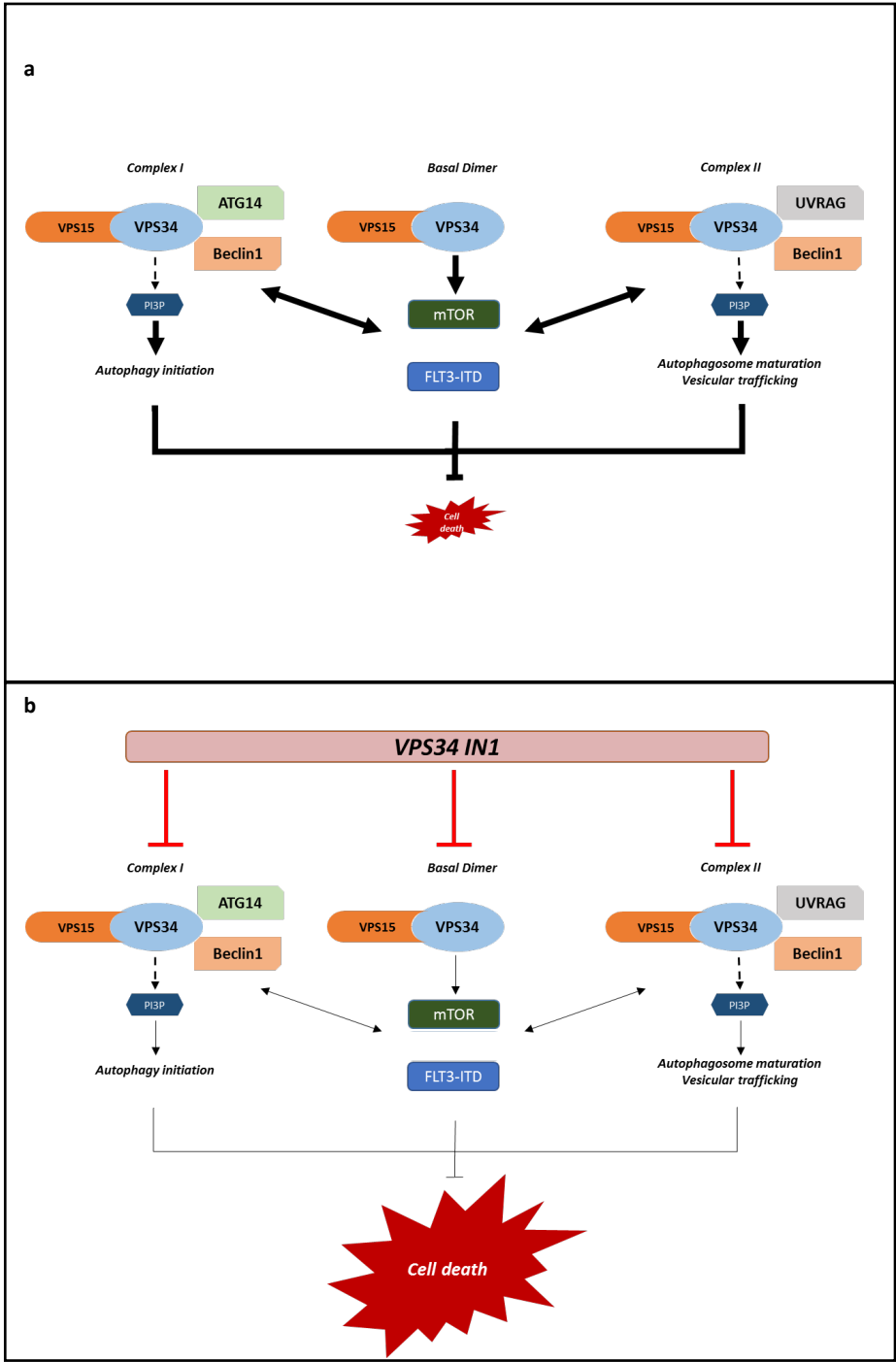

Supplemental Figure 9

Supplement: Supplementary file 14 — Supplemental Figure 9 [file 41389_2020_278_MOESM14_ESM.pdf]
