# Supplementary material for: Antileukemic activity of the VPS34-IN1 inhibitor in acute myeloid leukemia
Source: Oncogenesis. 2020 Oct 22;9(10):94. doi: 10.1038/s41389-020-00278-8 (PMC7581748; doi:10.1038/s41389-020-00278-8)
Supplement: Supplementary file 11 — Supplemental Figure 6 [file 41389_2020_278_MOESM11_ESM.pdf]

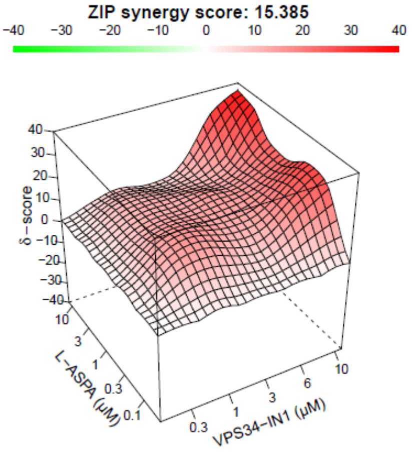

AML#21

VPS34-IN1 (µM)						
10	62	28	23	22	3	3
6	74	46	40	33	30	12
3	83	61	51	42	36	38
1	88	63	56	51	43	45
0,3	99	73	66	60	50	50
0	100	70	67	57	49	46
	0	*0,1*	*0,3*	*1*	*3*	*10*

L-Asparaginase (UI/ml)

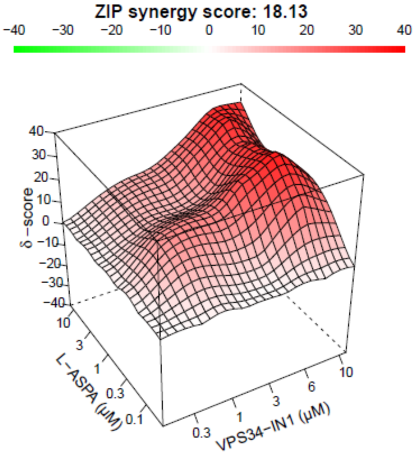

AML#22

VPS34-IN1 (µM)						
10	65	43	38	35	34	35
6	90	57	46	42	41	43
3	96	77	57	64	55	57
1	98	77	69	67	65	66
0,3	99	75	71	73	72	73
0	100	77	78	69	70	71
	0	*0,1*	*0,3*	*1*	*3*	*10*

L-Asparaginase (UI/ml)

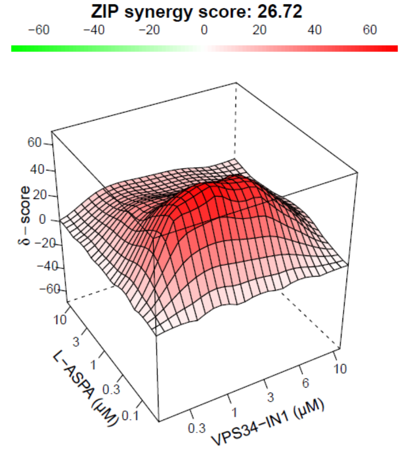

AML#23

VPS34-IN1 (µM)						
10	19	9	9	5	1	-1
6	27	12	11	8	5	2
3	38	15	12	7	3	0
1	64	33	22	7	4	0
0,3	78	44	26	12	8	1
0	100	95	94	74	42	13
	0	*0,1*	*0,3*	*1*	*3*	*10*

L-Asparaginase (UI/ml)

Supplemental Figure 6

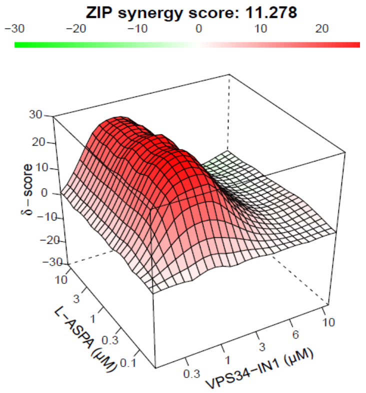

AML#24

VPS34-IN1 (µM) \ L-Asparaginase (UI/ml)	0	0,1	0,3	1	3	10
10	57	37	26	13	11	6
6	58	41	26	10	9	3
3	70	49	33	12	10	5
1	86	67	45	15	11	5
0,3	89	70	51	18	10	6
0	100	93	76	23	12	7

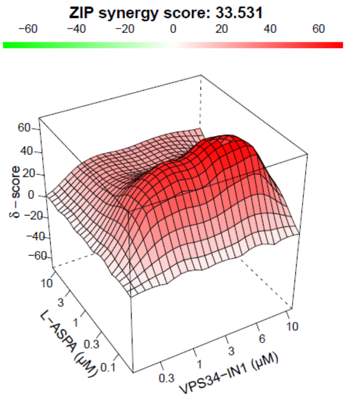

AML#25

VPS34-IN1 (µM) \ L-Asparaginase (UI/ml)	0	0,1	0,3	1	3	10
10	45	24	18	17	14	10
6	45	24	19	18	18	12
3	56	26	20	19	15	9
1	96	51	33	25	32	16
0,3	105	58	45	38	40	15
0	100	102	95	100	95	35

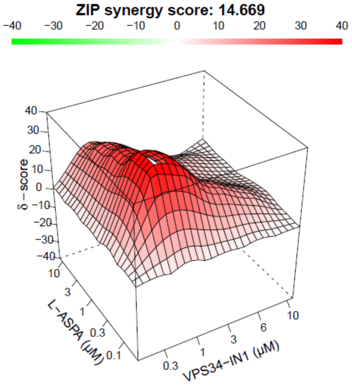

AML#26

VPS34-IN1 (µM) \ L-Asparaginase (UI/ml)	0	0,1	0,3	1	3	10
10	58	29	34	23	11	3
6	59	40	39	33	20	10
3	62	38	27	39	10	2
1	70	37	31	23	20	4
0,3	85	41	36	25	13	4
0	100	81	67	50	22	5

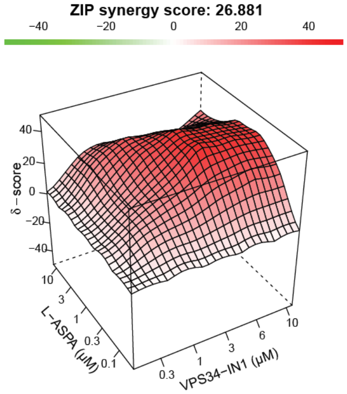

AML#27

VPS34-IN1 (µM) \ L-Asparaginase (UI/ml)	0	0,1	0,3	1	3	10
10	53	32	27	24	25	26
6	72	49	36	27	25	27
3	93	71	52	34	31	35
1	99	83	62	38	35	37
0,3	105	91	84	60	49	52
0	100	96	92	81	75	76

Supplemental Figure 6
